# Fulminant Guillain–Barré syndrome developed after surgical treatment of intracranial hemorrhage due to arteriovenous malformation: a case report

**DOI:** 10.1007/s10072-021-05384-y

**Published:** 2021-07-29

**Authors:** Hongyuan Liu, Liling Yang, Zongping Li, Gang Cheng

**Affiliations:** 1 Department of Neurosurgery, Mianyang Central Hospital, School of Medicine, University of Electronic Science and Technology of China, Mianyang, Sichuan People’s Republic of China; 2 Department of Nephrology, Mianyang Central Hospital, School of Medicine, University of Electronic Science and Technology of China, Mianyang, Sichuan People’s Republic of China

**Keywords:** Intracranial arteriovenous malformations, Cerebral hemorrhage, Guillain–Barré syndrome

## Abstract

Guillain–Barré syndrome (GBS) is a rare autoimmune disorder. GBS after surgical treatment of intracranial hemorrhage due to arteriovenous malformation (AVM) is even rarer. We present a 62-year-old man diagnosed with intracranial AVM and cerebral hemorrhage. He developed GBS after the operation for AVM and cerebral hemorrhage. Following surgical excision of AVM and cerebral hematoma, the patient developed generalized weakness, with subsequent quadriplegia and life-threatening dyspnea. The diagnosis was confirmed to be the acute motor–sensory axonal neuropathy subtype of GBS after cerebrospinal fluid analysis and antibody tests. The patient responded poorly to immunoglobulin and steroid therapy. His family abandoned further management and signed out of the hospital against medical advice. Despite being rare, GBS can occur after intracranial hemorrhage and surgery. Clinicians should rule out GBS when patients show no improvement or develop new neurologic

## Introduction

Guillain–Barré syndrome (GBS) is an autoimmune neuropathy characterized by the demyelination of peripheral nerves and nerve roots. The dysregulated immune response is usually triggered by infections, such as respiratory or gastrointestinal illness[1]. GBS has also been reported following immunization, bone marrow transplantation, or after cerebral hemorrhage, although rare[2]. Intracranial arteriovenous malformation (AVM), resulting in intracerebral hemorrhage with subsequent GBS, has never been reported. Here, we present a patient with fulminant GBS following surgical treatment of AVM and cerebral hematoma.

## Case presentation

Written informed consent was obtained from the wife of the patient for the publication of this case report and any accompanying images.

**Fig. 1:**
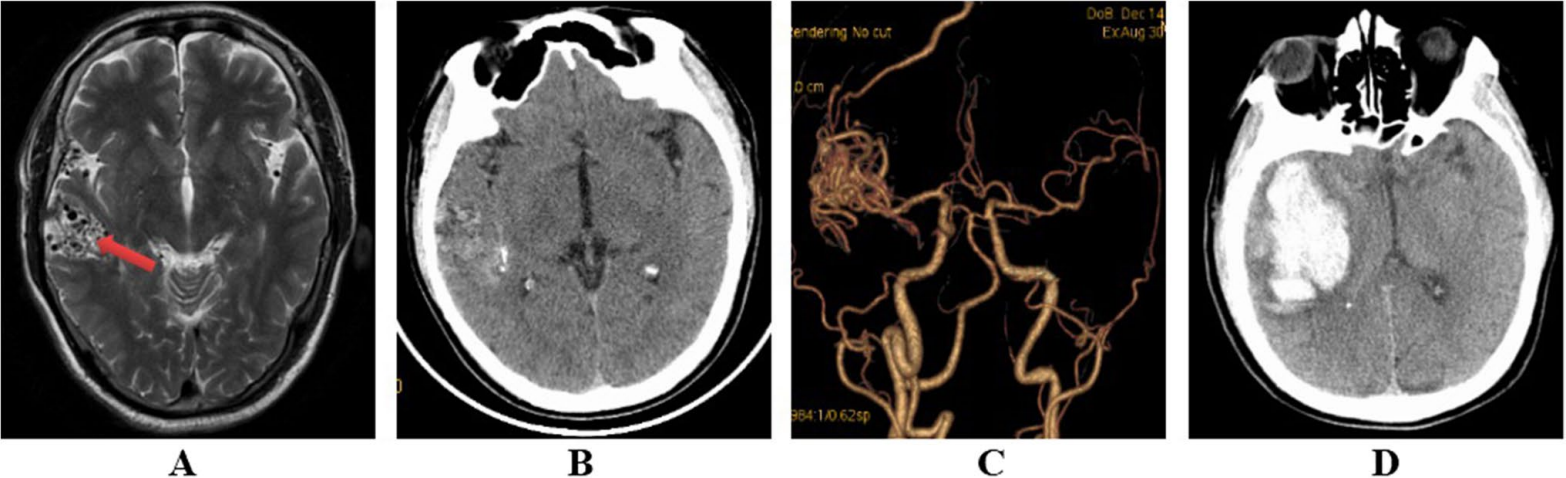
**A** MRI in 2018 revealed multiple tortuous and thickened blood vessels in the right temporal lobe. **B**, **C** CT angiography in 2018 showed clusters of multiple twisted and thickened vessels draining into the venous sinuses in the temporal lobe. **D** CT scan during the current hospital admission showed a large hematoma in the right temporal and insular lobe

A 62-year-old man presented with sudden left extremity weakness and vomiting for four hours on November 26, 2019. In 2018, because of left eyelid insufficiency and angular askew, computerized tomography (CT) angiography, magnetic resonance imaging (MRI), lumbar puncture, and electromyography were performed for the diagnosis of left facial neuritis and AVM (Fig. 1A–C). Left facial neuritis was cured, but the patient never received any treatment for AVM. No significant change in AVM was observed in the dynamic CT angiography review in 2018–2019. The wife also reported that the patient had a history of gastric ulcers and took unknown medications. An emergent head CT scan revealed a massive cerebral hemorrhage in the right temporal and insular lobes (Fig. 1D). The patient was diagnosed with right temporal lobe AVM associated with intracerebral hemorrhage.

On admission, the vital signs for this patient were as follows: temperature of 36.4 °C, blood pressure of 150/82 mmHg, heart rate of 66 beats/min, respiratory rate of 18 breaths/min, and oxygen saturation of 99%. He was lethargic, with left extremity weakness (muscle strength: 3/5 in the left extremities and 5/5 in the right extremities) but the normal sensation on examination. Blood tests showed a high white blood cell (WBC) count of 20.73*10^9^/L (normal reference: 3.5–9.5*10^9^/L), with a normal platelet count, procalcitonin level, and coagulation functions. His AVM was excised, and the intracranial hematoma was removed successfully by the emergent operation.

**Fig. 2:**
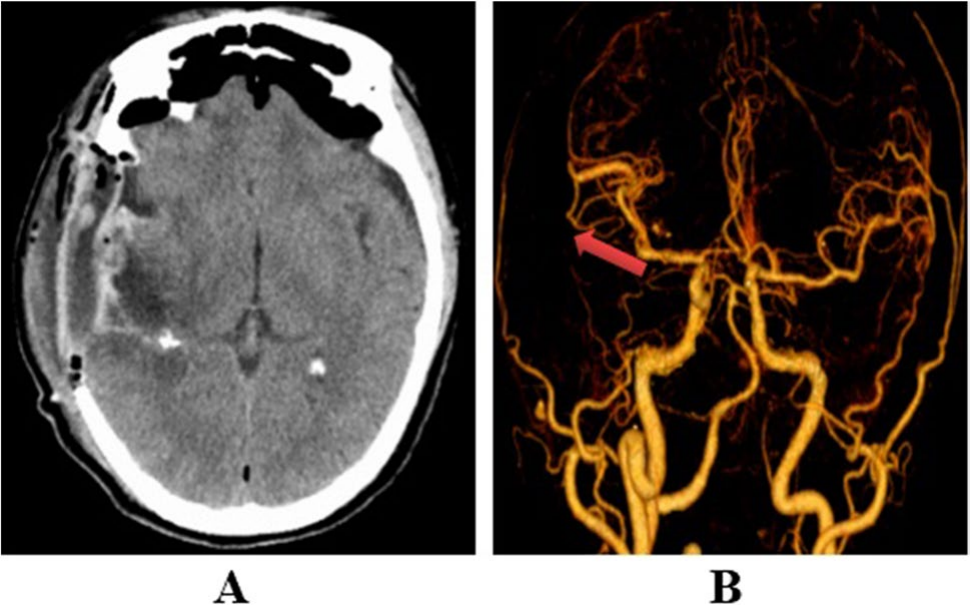
**A** The hematomas in the right temporal and insular lobe were removed. **B** The AVM in the temporal lobe was removed

After the operation, he was admitted to the medical floor and received prophylactic antibiotics, acid-suppressive medication, anticonvulsants, and supportive care. The patient gradually became more awake, but his left extremity muscle strength showed no significant improvements. Repeat post-operative head CT angiography showed that the AVM and hematoma were removed (Fig. 2). On the eighth day after the operation, the patient reported weakness in the bilateral extremities, which rapidly developed into bilateral extremity paralysis (muscle strength: 0/5 in four extremities) without pain and warmth sensations as well as the absence of deep tendon reflex. He then had severe dyspnea with reduced oxygen saturation to 89%. The patient was immediately transferred to the intensive care unit (ICU) for endotracheal intubation and ventilator support.

In the ICU, the patient was conscious with normal vital signs. Head CT angiography and cervical spine CT scans were performed. There were no signs of recurrent cerebral hemorrhage, infarction, or cervical spondylosis. Laboratory tests also reported normal WBC count, serum sodium, and procalcitonin level. Repeated sputum cultures did not report any bacteria. On the ninth day after the initial operation, cerebrospinal fluid (CSF) analysis revealed a nucleated cell count of 25*10^6^/L (normal reference: 0–6*10^6^/L), a protein concentration of 1020 mg/L (normal reference: 80–430*10^6^/L), and a negative bacterial culture. Immunoglobulin G and M assessments of anti-GM1 in the blood and CSF were positive. Bedside electromyography was not available prior to the initiation of the treatments.

The patient was treated with human immunoglobulin (0.4 g/kg/day) for 5 days and methylprednisolone (500 mg/day) for 3 days. There were no changes in his muscle strength or sensory functions. Tracheotomy was performed, and assisted ventilation was continued. Plasmapheresis anticoagulants have bleeding risks, and his wife refused plasmapheresis. Immunoglobulin and hormones were administrated for another 5 days. His extremity muscle strength improved slowly and reached the pre-operative level, but he could not wean from the ventilator. Finally, the patient and his family decided to stop the treatments. They signed out of the hospital against medical advice.

## Discussion

GBS is an inflammatory peripheral neuropathy mainly associated with infections from *Campylobacter jejuni*, cytomegalovirus, and Epstein–Barr virus. GBS can be classified into four subtypes including acute inflammatory demyelinating polyneuropathy (AIDP), acute motor axonal neuropathy (AMAN), Miller–Fisher syndrome (MFS), and acute motor–sensory axonal neuropathy (AMSAN)[3]. Among these, AIDP is the most common subtype, with mononuclear macrophages directly attacking the myelin proteins to destroy the myelin sheath. Meanwhile, an elevated ganglioside antibody concentration can often be detected in the AMAN and AMSAN subtypes of GBS. In the present case, on the eighth day after surgery, the patient had decreased muscle strength in the four extremities with sensory impairments, dyspnea, and CSF protein-cell separation. Antibodies against ganglioside were detected in the CSF and blood. In combination with the Chinese guidelines for diagnosis and treatment of Guillain–Barré 2019, this patient was considered as AMSAN[4]. The pathogenesis of GBS is not clear, but the infections in the respiratory and gastrointestinal tracts, bone marrow transplantation, vaccination, and surgery were reported to happen prior to its occurrence.

Infections in the respiratory and gastrointestinal tracts due to bone marrow transplantation, vaccination, and surgical operations may induce the immune response of the body to produce cross-reactions between antibodies and ganglion glycosides, with subsequent demyelination of the peripheral nerves. In the present case, no vaccination was administered before the surgery. There was no reported symptom to suggest respiratory or gastrointestinal tract infections before the incidence. A high pre-operative WBC count was considered due to the stress experienced after the intracerebral hemorrhage. The WBC count and procalcitonin level were normal after the operation and the sputum culture was also negative. Therefore, the possibility of GBS associated with respiratory or gastrointestinal tract infections or vaccination was excluded. However, the patient had a history of facial neuritis in 2018. The clinical manifestations of atypical GBS could be the same as partial facial neuritis. Although lumbar puncture and electromyography results in 2018 did not support GBS, caution is still needed.

It is reported that surgical operations, mostly orthopedic and gastrointestinal procedures, may induce GBS[5]. GBS associated with intracerebral hemorrhage is rarely reported. GBS might be caused by the destruction of the blood–brain barrier and the production of ganglioside antibodies induced by exogenous ganglioside with subsequent immune attacks on the peripheral nerves[6]. It has never been reported that GBS was induced by VAM with intracerebral hemorrhage. ASMAN following intracerebral hemorrhage and surgery has also never been reported. VAM with intracranial hemorrhage can disrupt the blood–brain barrier. Surgery can further increase the damage to the blood–brain barrier, making it easier for the immunogenic substance to enter the CSF circulation, which results in ganglioside antibody production and nerve demyelination. The ganglioside antibody can be detected in the blood and CSF.

Patients diagnosed with severe GBS should receive a continuous intravenous infusion of immunoglobulin or plasma exchange as soon as possible. However, the efficacy of immunoglobulin in combination with plasma exchange therapy is not superior to plasma exchange alone or immunoglobulin therapy alone. For patients who do not respond well to the first treatment course, additional immunoglobulin therapy should be attempted[7]. Our patient was critically ill and received immunoglobulin combined with glucocorticoids once his diagnosis of GBS was confirmed. He responded poorly to the treatments and finally gave up further management.

## Conclusion

GBS can occur after intracranial disorder or surgery. Its diagnosis can be easily missed since the symptoms may be masked by the presentations from the primary illness. Therefore, clinicians should rule out GBS when patients show no improvement or develop new neurological symptoms after the appropriate treatments for the primary disorder. Immunoglobulin and plasma exchange are the mainstay treatments for GBS and should be initiated once the diagnosis of GBS is confirmed.
